# Immortalisation of human oesophageal epithelial cells by a recombinant SV40 adenovirus vector.

**DOI:** 10.1038/bjc.1995.158

**Published:** 1995-04

**Authors:** S. Inokuchi, H. Handa, T. Imai, H. Makuuchi, M. Kidokoro, H. Tohya, S. Aizawa, K. Shimamura, Y. Ueyama, T. Mitomi

**Affiliations:** Department of Critical and Emergency Medicine, Tokai University School of Medicine, Kanagawa, Japan.

## Abstract

**Images:**


British Journal of Cancer (1995) 71, 819-825
© 1995 Stockton Press All rights reserved 0007-0920/95 $12.00

### Immortalisation of human oesophageal epithelial cells by a recombinant SV40 adenovirus vector

S Inokuchi[1,2], H Handa[3], T Imai[3], H Makuuchi[4], M Kidokoro[1], H Tohya[2], S Aizawa[5], K Shimamura[2,6], Y Ueyama[2,6], T Mitomi[4] and Y Sawada[1]

[1]*Department of Critical and Emergency Medicine, Tokai University School of Medicine;* [2]*Kanagawa Academy for Science and Technology;* [3]*Faculty of Bioscience and Biotechnology, Tokyo Institute of Technology;* [4]*Department of Surgery, Tokai University School of Medicine;* [5]*Department of Internal Medicine, Tokyo Medical College;* [6]*Department of Pathology, Tokai University School of Medicine.*

**Summary** We introduced the origin-defective SV40 early gene into cultured human oesophageal epithelial cells by infection of a recombinant SV40 adenovirus vector. The virus-infected cells formed colonies 3-4 weeks after infection in medium containing fetal calf serum. When the cells derived from 'serum-resistant' colonies were then maintained in the serum-free medium with a low calcium ion concentration, some of them passed the cell crisis and kept growing for over 12 months. These cells, regarded as immortalised cells, resembled the primarily cultured oesophageal epithelial cells in morphology and had some of their original characteristics. Treatment of the cells with a high calcium concentration induced phenotypic changes. These cells still responded to transforming growth factor beta. When the immortalised cells were injected into severe combined immunodeficient mice, they transiently formed epithelial cysts, although the typical differentiation pattern of the oesophageal epithelium was not observed. These cysts regressed within 2 months without development into tumours. The results indicated that human oesophageal epithelial cells were reproducibly immortalised by infection with a recombinant SV40 adenovirus vector at relatively high efficiency. The immortalised cells should be useful in studies on oesophageal carcinogenesis and in assessing the cooperative effects with other oncogene products or carcinogens.

**Keywords:** human oesophageal epithelial cells; SV40 T antigen; recombinant SV40 adenovirus vector; transformation; immortalisation

The malignant transformation of human cells has been considered as a multistep phenomenon, although the mechanism of human carcinogenesis has not been fully elucidated (Peto *et al.*, 1975; Hunter, 1991). This concept is also supported by studies on neoplastic conversion of normal human cells *in vitro* (Rhim *et al.*, 1985, 1986, White *et al.*, 1992). In these studies, human cells immortalised by transfection of one viral oncogene did not show tumorigenecity *in vivo*. However, cooperation of a second oncogene or chemical carcinogen induced neoplastic properties in the immortalised cells.

While some genetic abnormalities of oesophageal cancer including mutation of P53 and overexpression or gene amplification of epidermal growth factor (EGF) receptor, int-2/hst-1 or cyclin D have been reported, the role of these abnormalities in oesophageal carcinogenesis is still unclear (Lu *et al.*, 1988; Hollstein *et al.*, 1990; Boyton *et al.*, 1991; Tsuda *et al.*, 1991; Jiang *et al.*, 1992; Wang *et al.*, 1993). It is considered that the immortalisation of oesophageal epithelial cells by viral oncogene facilitates the study of neoplastic transformation of the cells *in vitro*.

Gene transfer of foreign genes by the original calcium phosphate co-precipitation method (Graham and Van der Eb, 1973) is not suitable for human oesophageal epithelial cells because the cells undergo terminal differentiation by treatment with high concentrations of calcium. Although successful gene transfer and immortalisation of human oesophageal epithelial cells were performed by the strontium phosphate transfection method (Stoner *et al.*, 1991), the immortalisation efficiency was low. It is still difficult to immortalise human oesophageal epithelial cells reproducibly. We previously reported that infection with a recombinant SV40 adenovirus vector is an alternative method of gene transfer into human epidermal keratinocytes (Inokuchi *et al.*, 1991).

Correspondence: S Inokuchi, Department of Critical and Emergency Medicine, Tokai University School of Medicine, Bohseidai, Isehara, Kanagawa 259-11, Japan
Received 20 April 1994; revised 16 November 1994; accepted 22 November 1994

In this study, we introduced the origin-defective SV40 early gene into primarily cultured human oesophageal epithelial cells by a recombinant SV40 adenovirus vector and transformed or immortalised the infected cells. We show here that the virus vector was useful for the transfer of foreign genes into human oesophageal epithelial cells with relatively high efficiency. The SV40 T antigen conferred on oesophageal epithelial cells the ability to grow continuously and form colonies in the presence of serum. The cells derived from 'serum-resistant' colonies were maintained in serum-free medium with a low concentration of calcium ions. Some of them passed the cell crisis and kept growing under these conditions for over 12 months. Considered to be immortalised, they resembled the primarily cultured human oesophageal epithelial cells in morphology, and had some of the original characteristics of human oesophageal epithelial cells. They were not tumorigenic when injected into severe combined immunodeficient (SCID) mice.

### Materials and methods

### *Recombinant SV40 adenovirus vector*

The recombinant SV40 adenovirus, *ori*$^-$, was used in this study (Van Doren and Gluzman 1984). The origin-defective SV40 early gene was cloned into the adenovirus vector, delta-E1/X (Van Doren *et al.*, 1984), in place of the E1 region of the adenovirus. The *ori*$^-$ was able to propagate in a cell line, 293 cells (Graham *et al.*, 1977), expressing adenovirus E1a and E1b gene products. These 293 cells were maintained in Dulbecco's modified minimum essential medium (DMEM) supplemented with 10% fetal calf serum (FCS; JRH, Lenexa, CS, USA). When the 293 cells were subconfluent, the medium was removed and the cells were infected with *ori*$^-$ at multiplicity of infection (m.o.i.) of 0.1 plaque-forming units (PFUs) per cell after washing with serum-free DMEM. The infected cells were cultured in DMEM supplemented with 2% FCS until a complete adenovirus-specific cytopathic effect appeared. The culture medium including the infected cells was frozen and thawed four times followed by centrifugation at 3000 r.p.m. for 15 min. The supernatant was used as virus stock of *ori*⁻. The titre of *ori*⁻ was approximately $2 \times 10^8$ PFU $ml^{-1}$.

Culture medium containing virus-free 293 cells was also frozen and thawed. After centrifugation, the supernatant was used for mock virus infection.

### *Primary culture of human oesophageal epithelial cells*

Human oesophageal epithelial tissues were obtained from biopsies or surgical specimens with the informed consent of patients. After treatment with 2000 U $ml^{-1}$ Dispase II (Godo Shusei, Tokyo, Japan) at 37°C for 30 min and 0.5% trypsin solution at 37°C for 5 min, the epithelial cells were disaggregated by gently pipetting. The cells were propagated in growth medium, MCDB 153 (Clonetics, Mountain View, CA, USA) containing 0.1 mM calcium chloride, 140 $\mu g\ ml^{-1}$ bovine pituitary extract (BPE; Clonetics), 0.5 $\mu g\ ml^{-1}$ hydrocortisone, 0.1 ng $ml^{-1}$ human epidermal growth factor (h-EGF; Earth Pharmaceutical, Hyogo, Japan), 5 $\mu g\ ml^{-1}$ insulin, 0.1 mM phosphoethanolamine, 0.1 mM ethanolamine and 100 ng $ml^{-1}$ cholera toxin (List Biological, Campbell, CA, USA). The cells were passaged using a routine trypsinisation technique. Cells subcultured three times were used for infection of the virus vector. In the subcultured cells, no fibroblasts were identified by phase-contrast microscopy. When primary cultured oesophageal epithelial cells were continuously propagated in MCDB 153 growth medium, the cells ceased to grow after the fourth or fifth passage and gradually became detached from the dish. No transformed cells arose within 6 months.

### *Infection of the virus vector*

Cultured human oesophageal epithelial cells in a 60 mm dish were washed with phosphate-buffered saline (PBS), and then the virus stock was added at the indicated virus to target cell ratio (m.o.i.; multiplicity of infection). After 1 h incubation at 37°C for virus adsorption, the infected cells were washed and incubated in MCDB 153 growth medium at 37°C.

### *Immunocytochemical staining for SV40 T antigen*

The infected cells were fixed with methanol at 4°C for 30 min and stained for the SV40 T antigen by the indirect immunoperoxidase method using mouse monoclonal antibody against the SV40 T antigen (PAb 419; Oncogene Science, Manhasset, NY, USA) and horseradish peroxidase (HRP)-conjugated goat anti-mouse IgG. The enzyme reaction was performed with 0.02% 3,3′-diaminobenzidine (DAB) containing 0.005% hydrogen peroxide for 2 min.

### *Formation of 'serum-resistant' colonies by infection with* ori⁻

After infection with *ori*⁻, the oesophageal epithelial cells were cultured in the growth medium for a week and the culture medium was replaced with DMEM supplemented with 10% FCS. Four weeks after infection, colonies composed of continuously proliferating cells, 'serum-resistant' colonies, were scored. Normal oesophageal epithelial cells were terminally differentiated to stop growth and detached from the dishes under the culture conditions.

### *Immortalisation of oesophageal epithelial cells infected with* ori⁻

The cells of each 'serum-resistant' colony formed in DMEM supplemented with 10% FCS 4 weeks after infection were picked up and also subcultured in MCDB 153 growth medium without FCS. The cells which passed the crisis and kept growing in the growth medium for over 12 months were considered to be immortalised. Characteristics of one of the immortalised cells (HESSV 5-3) were examined. We also analysed the morphology of the cells, keratin production, sensitivity to human transforming growth factor beta (TGF β; Takara Shuzo, Kyoto, Japan) and their morphogenesis when they were injected into severe combined immunodeficient (SCID) mice (*CB17 scid/scid*).

### *Immunocytochemical staining*

Immortalised cells were stained for SV40 T antigen as described above. The cells were counterstained with 0.2% light green SFY (Wako Pure Chemical Industries, Osaka, Japan). Indirect immunostaining for human epithelial keratin was also performed using a monoclonal anti-keratin antibody (AE1; Boehringer-Mannheim Biochemicals, Indianapolis, IN, USA).

Both normal and immortalised cells were incubated for 24 h in MCDB 153 growth medium or medium containing 2 mM calcium, 10% FCS or 10 ng $ml^{-1}$ 12-*O*-tetradecanoyl phorbol-13-acetate (TPA). After fixation with acetone at − 20°C for 20 min, the cells were incubated in rabbit anti-involucrin antibody (Biotechnologies, Stouton, MA, USA), followed by treatment with fluorescein-conjugated anti-rabbit antibody. Involucrin-positive cells were analysed by light microscopy.

### *Extraction and analysis of keratins*

To prepare keratins, normal and immortalised oesophageal epithelial cells were sonicated in buffer A containing 20 mM Tris–HCl (pH 7.4), 0.6 M potassium chloride 1% Triton X-100 and 0.3 $\mu g\ ml^{-1}$ phenylmethylsulphonyl fluoride (PMSF) and then centrifuged at 10 000 r.p.m. for 20 min according to the method described previously (Moll *et al.*, 1992). The precipitates were suspended in buffer A, sonicated again followed by centrifugation. This step was repeated three more times. Final precipitates were solubilised in buffer B containing 20 mM Tris–HCl (pH 7.4), 2% sodium dodecyl sulphate (SDS), 10 mM dithiothreitol (DTT) and 8 M urea at 37°C for 30 min. An aliquot of the sample was subjected to two-dimensional gel electrophoresis (O'Farrell, 1975; Moll *et al.*, 1982). The gel was stained with Coomassie brilliant blue.

### *Effect of TGF-β on cellular DNA synthesis*

Normal and immortalised oesophageal epitheal cells ($2 \times 10^4$ cells per well) were cultured in a 24-well dish in MCDB153 growth medium containing the indicated amount of TGF-β for 24 h. The cells were pulse labelled with [$^3$H]-thymidine (37 KB per well) for 1 h. After washing with PBS three times, the cells were fixed with trichloroacetic acid (TCA) and the TCA-insoluble count was obtained according to the method described by Shipley *et al.* (1984). The [$^3$H]thymidine uptake per cell was calculated on the basis of the number of parallel cultured cells counted.

### *Proliferation and differentiation of immortalised oesophageal epithelial cells in SCID mice*

The cells derived from 'serum-resistant' colonies were cultured in the MCDB 153 growth medium. Approximately $1 \times 10^7$ normal oesophageal epithelial cells, virus-infected cells cultured for 1.5 months and immortalised cells were subcutaneously injected into SCID mice. Two weeks after injection, a nodule formed by the injected cells was excised from the mice and fixed with 10% formaldehyde solution. The structure of the cyst was examined by light microscopy after haematoxylin–eosin (H&E) staining.

### Results

### *Transient expression of SV40 T antigen in oesophageal epithelial cells infected with* ori⁻

Cultured human oesophageal epithelial cells were infected with *ori*⁻ at the indicated m.o.i. Expression of SV40 T

antigen in the infected cells was analysed by immunocytochemical staining. Table I shows that the SV40 T antigen-positive cells increased in number as the m.o.i. was increased. Between 15% and 30% of the infected cells were positive at an m.o.i. of 100 PFUs per cell.

*'Serum-resistant' colony formation*

Normal oesophageal epithelial cells differentiated and stopped growing in medium supplemented with serum. To examine whether the *ori*⁻-infected cells formed 'serum-resistant' colonies, primarily cultured oesophageal epithelial cells were infected with *ori*⁻ at the indicated m.o.i. The virus-infected cells were cultured in the MCDB 153 growth medium for a week. Then medium was changed to DMEM supplemented with 10% FCS and cultured for a further 3 weeks. These culture conditions were also used to select partially transformed murine keratinocytes (Yuspa and Morgen, 1981) and human papillomavirus (HPV)- or SV40-transformed human epidermal keratinocytes (Shlegel *et al.*, 1988; Inokuchi *et al.*, 1991). The medium was changed every 3 days. During the cultivation, almost all cells stopped growing and became detached from the dish. A few cells kept growing and formed 'serum-resistant' colonies. Table II shows the number of 'serum-resistant' colonies formed under these conditions. More colonies were formed as the m.o.i. was increased. Frequency of 'serum-resistant' colony formation was approximately 0.01% at an m.o.i. of 100 PFUs per cell. The colonies were analysed by light microscopy. Figure 1 shows that the 'serum-resistant' colony was composed of many small cells and a few larger cells.

**Table I** Transient expression of SV40 T antigen after infection with recombinant virus vector

| *Multiplicity of infection (m.o.i.)* | *Percentages of T antigen positive cells*[a] |
|---|---|
| 100 | 29.0, 16.6 |
| 10 | 4.3, 3.2 |
| 1 | 1.4, 1.0 |
| Mock[b] | <0.1, <0.1 |

[a]Cells were fixed and stained 48 h after infection. [b]Treated with virus-free supernatant of 293 cells.

**Table II** 'Serum-resistant' colony formation after infection with recombinant virus vector

| *Multiplicity of infection (m.o.i.)* | *Number of 'serum-resistant' colonies (per $10^5$ cells)*[a] |
|---|---|
| 100 | 16, 9 |
| 10 | 4, 1 |
| 1 | 1, 0 |
| Mock | 0, 0 |

[a]Number of 'serum-resistant' colonies 4 weeks after infection.

**Figure 1** Morphology of a 'serum-resistant' colony 4 weeks after infection of the recombinant SV40 adenovirus vector examined by phase-contrast microscopy. Bar = 200 μm.

*Immortalisation of the cells infected with* ori⁻

The cells in each 'serum-resistant' colony described above were picked up using cloning cylinders and were subcultured in MCDB 153 growth medium in the absence of FCS. The subcultured cells were passaged when they became confluent in a dish. The SV40 T antigen was expressed in all cells in 'serum-resistant' colonies to the extent that they were examined. However, the majority of cells underwent the crisis within 2 months after subculture in MCDB 153 growth medium without serum. During the crisis period, almost all of the cells were still immunopositive for SV40 T antigen. After long crisis periods, some of the cells derived from 'serum-resistant' colonies passed the crisis and kept growing for over 12 months under the culture conditions described. These cells expressed both SV40 T antigen (Figure 2a) and human epithelial keratin (Figure 2b). The cells exhibited a small cobblestone pattern (Figure 3b) and resembled primarily cultured oesophageal epithelial cells (Figure 3a) in morphology. Therefore, they were regarded as immortalised oesophageal epithelial cells. Table III shows the number of colonies from which cells were immortalized.

In contrast, when the cells derived from 'serum-resistant' colonies were passaged into DMEM containing 10% FCS after treatment with trypsin, the cells always differentiated and stopped growing. Immortalised cells did not appear. These results suggested that the cells were not completely insensitive to FCS, even though they were derived from 'serum-resistant' colonies.

**Figure 2** Immunocytochemical staining of immortalised cells for SV40 T antigen (**a**) and human keratin (**b**). The cells were counterstained with 0.2% light green SF (**a**).

Figure 3 Morphology of normal oesophageal epithelial cells (a) and immortalised oesophageal epithelial cells cultured in MCDB 153 growth medium containing 0.1 mM calcium (b). Immortalised cells grown in medium containing 2 mM calcium for 48 h (c). Phase-contrast microscopy. Bar = 100 μm.

### Characterisation of the immortalised cells

Normal oesophageal epithelial cells cultured in MCDB 153 growth medium were altered morphologically when the calcium concentration was increased to 2 mM. The alteration was associated with cell differentiation *in vitro*. Therefore, we examined the effect of calcium ions on the immortalised cells. The immortalised cells were cultured in growth medium with 2 mM calcium ions. Figure 3b and c shows that high calcium concentration induced morphological alteration of the immortalised cells. When the immortalised cells were cultured in growth medium with low calcium concentration (0.1 mM), cells homogeneous in size formed a cobblestone monolayer with loose contact between the cells (Figure 3b). However, 48 h after an increase in concentration of calcium ions to 2 mM, the cell morphology was altered and became flattened with relatively close cell-to-cell contact (Figure 3c).

It has been reported that some agents induce terminal differentiation of cultured human epidermal keratinocytes (Mufson *et al.*, 1982; Pillai *et al.*, 1988). To examine the effect of some of the inducers on differentiation of normal or immortalised oesophageal epithelial cells, we analysed the

**Table III** Immortalisation of cells derived from 'serum-resistant' colonies

| *Sample no.* | *Number of 'serum-resistant' colonies picked up*[a] | *Immortalised colonies*[b] |
|---|---|---|
| 1 | 3 | 0 |
| 2 | 2 | 0 |
| 3 | 3 | 0 |
| 4 | 6 | 0 |
| 5 | 10 | 2 |
| 6 | 2 | 1 |
| 7 | 5 | 0 |
| 8 | 2 | 0 |

[a]Number of 'serum-resistant' colonies formed when $10^5$ cells were infected with the recombinant virus at an m.o.i. of 10 PFUs per cell. [b]Number of immortalised colonies among the 'serum-resistant' colonies subcultured.

**Table IV** Percentage of involucrin-positive cells[a]

| | *Inducers*[b] | | | |
|---|---|---|---|---|
| | *None* | *10% FCS* | *2 mM $Ca^{2+}$* | *TPA* |
| Normal oesophageal epithelial cells | 2 | 14 | 16 | 15 |
| SV40-immortalised oesophageal epithelial cells | <0.1 | 0.2 | <0.1 | <0.1 |

[a]One thousand cells were counted for each value. [b]Cells were cultured in MCDB 153 with each inducer added for 24 h.

**Table V** Effect of TGF-β on cellular DNA synthesis[a]

| | *Concentration of TGF-$\beta_1$ (ng $ml^{-1}$)* | | | | |
|---|---|---|---|---|---|
| | *0* | *0.1* | *1* | *10* | *30* |
| Normal oesophageal epithelial cells | 100 | 68 | 36 | 15 | 18 |
| SV40-immortalised oesophageal epithelial cells | 100 | 103 | 81 | 76 | 60 |

[a]Percentage [$^3$H]thymidine uptake in treated cells with respect to the untreated cells. Each number is a mean value of triplicate experiments.

expression of involucrin after induction using immunocytochemical staining. Involucrin, one of the differentiation markers, is a precursor protein of the cornified envelope of human epidermis and is localised above the suprabasal layer in normal eosophageal epithelial tissues (Banks-Schlegel and Green, 1981). Table IV shows that involucrin-positive cells were increased in number when normal oesophageal epithelial cells were treated with high concentrations of calcium ions, FCS or TPA. However, such treatment hardly affected the immortalised cells.

Cellular DNA synthesis was blocked by treatment of TGF-β in normal human epidermal keratinocytes, but not in the SV40-transformed human epidermal keratinocytes (Shipley *et al.*, 1986; Pietenpol *et al.*, 1990). We examined the effect of TGF-β on DNA synthesis of normal or immortalised oesophageal epithelial cells 24 h after treatment. Table V shows that normal oesophageal epithelial cells were sensitive to TGF-β. Compared with the normal cells, the immortalised cells were less sensitive to TGF-β, but the cells still retained sensitivity.

It has been reported that SV40-transformed epidermal keratinocytes express fetal-type keratins (Bernard *et al.*, 1985; Morris *et al.*, 1985). To examine whether immortalised oesophageal epithelial cells expressed these proteins, keratin fractions were prepared from normal and immortalised human oesophageal epithelial cells and analysed as described in Materials and methods. Keratins with molecular masses of 45 kDa and 52.5 kDa were detected in the immortalised cells (Figure 4b) but not in cultured normal oesophageal epithelial cells (Figure 4a). In addition to these keratins, a spot with *c.*

**Figure 4** Two-dimensional gel electrophoresis of keratins. Keratins were prepared from cultured normal oesophageal epithelial cells (**a**) or immortalised cells (**b**). Arrowheads indicate no. 18 and 8 keratins with molecular masses of 45 and 52.5 kDa respectively.

60 kDa molecular mass and pI 5.4 was detected in the immortalised cells, but we have not yet characterised it.

### *Proliferation and differentiation of the immortalised oesophageal epithelial cells in SCID mice*

The oesophageal epithelial cells derived from 'serum-resistant' colonies cultured in the MCDB 153 growth medium for 1.5 months were termed precrisis cells. Either precrisis cells or immortalised cells were subcutaneously injected into SCID mice. Cultured normal eosophageal epithelial cells were also injected into the mice. All of them transiently formed epithelial cysts at the injection site 2 weeks after injection, which regressed within 2 months. No tumour was formed within 12 months after regression of the cyst, suggesting that the immortalised cells were not tumorigenic.

Histological analyses showed that both normal and 1.5 month cultured precrisis cells proliferated and differentiated in SCID mice. The normal cells generated cysts, in which fully differentiated oesophageal epithelium was reorganised (Figure 5a). After injection of the precrisis cells, an apparent basal layer and middle layer of the oesophageal epithelium with intercellular bridge formation were constructed in the interior of the cyst, although the upper layer was not fully organised (Figure 5b). The immortalised, post-crisis cells also proliferated and differentiated, but they did not show the typical differentiation pattern (Figure 5c). In this case, the basal layer was indistinct and the polarity of cell differentiation was obscure. Irregularities in nuclear size and shape were also observed.

**Figure 5** Photomicography of a cyst wall in SCID mice (H&E staining). Bar = 50μm. The cyst was formed 2 weeks after injection of normal oesophageal epithelial cells (**a**) and cells derived from a 'serum-resistant' colony which kept growing in MCDB 153 growth medium for 1.5 months (**b**) and for over 12 months (**c**).

### Discussion

Highly efficient SV40 early gene expression was obtained when human oesophageal epithelial cells were infected with a recombinant SV40 adenovirus vector. This indicated that a recombinant adenovirus vector should be useful to transfer foreign genes into human oesophageal epithelial cells as well as human epidermal keratinocytes as described previously (Inokuchi *et al.*, 1991).

Human oesophageal epithelial cells were transformed into 'serum-resistant' cells by SV40 T antigen, as were human epidermal keratinocytes (Steinberg and Defendi, 1979; Inokuchi *et al.*, 1991), although the original cells differentiated and stopped growing in the presence of serum. The frequency of 'serum-resistant' colony formation of human oesophageal epithelial cells infected with $ori^-$ at an m.o.i. of 100 PFUs per cell, 0.01%, was almost the same as that of human epidermal keratinocytes (Inokuchi *et al.*, 1991).

More 'serum-resistant' colonies were formed when higher m.o.i. values were employed (see Table II). However, colonies were so dense that each colony could not be separately picked up. Therefore, the $ori^-$-infected oesophageal epithelial cells were infected at an m.o.i. of 10 PFUs per cell in order to pick up 'serum-resistant' colonies. When cells picked up from the colonies were subcultured in the MCDB 153 growth medium, some of the 'serum-resistant' cells acquired the capacity to keep growing in the serum-free medium with low calcium concentration for over 12 months. These cells were considered to have passed the cell crisis and become immortalised. These immortalised cells developed from only three out of the 33 'serum-resistant' colonies (less than 10%). Such immortalisation was also observed in the 'serum-resistant' epidermal keratinocytes transformed by the SV40 early gene or human papillomavirus type 16 gene (Schlegel *et al.*, 1988; Inokuchi *et al.*, 1991). In this case, more than 80% of epidermal keratinocytes derived from 'serum-resistant' colonies were immortalised. This suggested that the immortalisation frequency of human oesophageal epithelial cells was lower than that of human epidermal keratinocytes. Although the exact reason is not known, it might be due to the difference in their original characteristics or the number of oncogenes introduced.

In our results, almost all of the cells derived from 'serum-resistant' colonies continuously expressed the SV40 T antigen. However, the majority of them showed limited lifespans and only a few cells passed the crisis period. These findings suggested that the expression of SV40 T antigen itself did not directly cause immortalisation of human oesophageal epithelial cells. Although the cells in 'serum-resistant' colonies were selected for both resistance to differentiation and prolonged cell growth, additional unknown event(s) appear to be required for their immortalisation.

SV40-transformed epidermal keratinocytes showed increased expression of keratin species with molecular masses of 45 kDa and 52.5 kDa, i.e. keratin nos. 18 and 8 respectively in Moll's catalogue (Moll *et al.*, 1982; Bernard *et al.*, 1985; Morris *et al.*, 1985). The immortalised oesophageal epithelial cells also expressed these keratins (see Figure 4b). This suggested that expression of these keratins was partly dependent on SV40 T antigen expression. These keratins were also found in fetal and simple epithelial cells (Moll *et al.*, 1982). However, they were only rarely detected in normal oesophageal epithelium or human eosophageal carcinoma (Moll *et al.*, 1982; Grace *et al.*, 1985; Banks-Schlegel and Quinto, 1986; Boch *et al.*, 1988).

The immortalised oesophageal epithelial cells preserved characteristics of normal oesophageal epithelial cells. The cells were altered morphologically by treatment with high calcium ion concentrations and were also sensitive to TGF-$\beta$, although they were less sensitive than the normal cells (see Table V). It was also reported that SV40-immortalised human esophageal epithelial cells had the ability to respond to TGF-$\beta$ (Stoner *et al.*, 1991). However, SV40-immortalised epidermal keratinocytes were usually found to be insensitive to TGF-$\beta$ (Pietenpol *et al.*, 1990). Therefore, the sensitivity of the SV40-immortalised cells to TGF-$\beta$ differed between human oesophageal epithelial cells and human epidermal keratinocytes. This suggested that the signal transduction pathways of TGF-$\beta$ through its receptor were different.

Primarily cultured oesophageal epithelial cells were induced to express involucrin by treatment with some differentiation inducers. However, the immortalised cells hardly expressed involucrin after treatment with the inducers under our assay conditions. This suggested that the immortalised cells lost their original capacity to respond to the inducers.

The cells derived from 'serum-resistant' colonies were maintained in the serum-free medium for 1.5 months as precrisis cells. When either the precrisis cells or the immortalised cells were subcutaneously injected into SCID mice, they transiently formed epithelial cysts at the injection site. However, they were not tumorigenic. The 1.5 months cultured precrisis cells formed epithelial structures with an apparent basal layer and a differentiation pattern of oesophageal epithelium. The 12 months cultured immortalised cells also formed a cyst. However, the inner structure was different from that formed by precrisis or normal oesophageal epithelial cells. The basal layer and cellular polarity were obscure. The results indicated that immortalised cells cultured for over 12 months did not retain all of the original activity of regeneration *in vivo*. However, precrisis cells cultured for 1.5 months still retained the ability to regenerate a structure resembling normal oesophageal epithelium, even though they were derived from the 'serum-resistant' colony.

The oesophageal epithelial cells immortalised by recombinant SV40 adrenovirus vector still had some of their original characteristics and were not tumorigenic in SCID mice. These immortalised cells should be useful in studying oesophageal carcinogenesis and in assessing cooperative effects with other oncogene products or carcinogens.

**Acknowledgements**
This work was supported by a Grant-in-Aid for Scientific Research from Tokai University to SI and a Grant-in-Aid for Cancer Research from the Ministry of Education, Science and Culture to HH.

### References

BANKS-SCHLEGEL SP AND GREEN H. (1981). Involucrin synthesis and tissue assembly by keratinocytes in natural and cultured human epithelia. *J. Cell Biol.*, **90**, 732–737.

BANKS-SCHLEGEL SP AND QUINTO J. (1986). Growth and differentiation of human esophageal carcinoma cell lines. *Cancer Res.*, **46**, 250–258.

BERNARD BA, ROBINSON SM, SEMAT A AND DARMON M. (1985). Reexpression of fetal characters in simian virus 40-transformed human keratinocytes. *Cancer Res.*, **45**, 1707–1716.

BOCH FX, LEUBE RE, ACHTSTATTER T, MOLL R AND FRANKE WW. (1988). Expression of simple epithelial type cytokeratins in stratified epithelia as detected by immunolocalization and hybridization in situ. *J. Cell Biol.*, **106**, 1635–1648.

BOYNTON FR, HUANG Y, BLOUNT LP, REID JB, RASKIND HW, HAGGITT CR, NEWKIRK C, RESEAU HJ, YIN J, MCDANIAL T AND MELTZER JS. (1991). Frequent loss of heterozygosity at the retinoblastoma locus in human esophageal cancer. *Cancer Res.*, **51**, 5766–5769.

GRACE MP, KIM KH, TRUE LD AND FUCHS E. (1985). Keratin expression in normal esophageal epithelium and squamous cell of the esophagus. *Cancer Res.*, **45**, 841–846.

GRAHAM R AND VAN DER EB A. (1973). A new technique for the assay of infectivity of human adenovirus 5 DNA. *Virology*, **52**, 456–467.

GRAHAM FL, SMILEY JS, RUSSEL WC AND NARIN R. (1977). Characteristics of a human cell line transformed by DNA from human adenovirus type 5. *J. Gen. Virol.*, **36**, 59–72.

HOLLSTEIN MC, METCALF RA, WELSH JA, MONTESANO R AND HARRIS CC. (1990). Frequent mutation of the p53 gene in human esophageal cancer. *Proc. Natl Acad. Sci. USA*, **87**, 9958–9961.

HUNTER T. (1991). Cooperation between oncogenes. *Cell*, **64**, 249–270.

INOKUCHI S, UEDA M, HASHIMOTO K, HANDA H AND MITOMI T. (1991). Immortalization of human epidermal keratinocytes by the recombinant SV40 adenovirus vector. In *Vitro Cell Dev. Biol.*, **27A**, 827–828.

JIANG WJ, KAHN SM, TOMITA N, ZHANG YJ, LU SH AND WEINSTEIN B. (1992). Amplification and expression of the human cyclin D gene in esophageal cancer. *Cancer Res.*, **52**, 2980–2983.

LU SH, HSIEH LL, LUO FC AND WEINSTEIN IB. (1988). Amplification of EGF receptor and c-myc genes in human esophageal cancers. *Int. J. Cancer*, **42**, 502–505.

MORRIS AM, STEINBERG ML AND DEFENDI V. (1985). Keratin gene expression in simian virus 40-transformed human keratinocytes. *Proc. Natl Acad. Sci. USA*, **82**, 8398–8402.

MOLL R, FRANKE WW, SCHILLER DL, GEIGER B AND KREPLER R. (1982). The catalog of human cytokeratins: patterns of expression in normal epithelia, tumors and cultured cells. *Cells*, **31**, 11–24.

MUFSON RA, STEINBERG ML AND DEFENDI V. (1982). The effects of 12-O-tetradecanoyl phorbol-13-acetate on the differentiation of SV40 infected human keratinocytes. *Cancer Res.*, **42**, 4600–4605.

O'FARRELL PH. (1975). High resolution two-dimensional electrophoresis of proteins. *J. Biol. Chem.*, **250**, 4007–4021.

PETO R, ROE FJC, LEE PN, LEVY L AND CLERK J. (1975). Cancer and aging in mice and men. *Br. J. Cancer*, **32**, 411–426.

PIETENPOL JA, STEIN RW, MORAN E, YACIUK P, SCHLEGEL R, LYONS RM, PITTELKOW R, MUNGER K, HOWLEY PM AND MOSES HL. (1990). TGF-beta 1 inhibition of c-myc transcription and growth in keratinocytes is abrogated by viral transforming proteins with pRB binding domains. *Cell*, **61**, 777–785.

PILLAI S, BILKE DD, HINCENBURGS M AND ELIAS PM. (1988). Biochemical and morphological characterization of growth and differentiation of normal human neonatal keratinocytes in a defined medium. *J. Cell Physiol.*, **134**, 229–237.

RHIM JS, JAY G, ARNSTEIN P, PRICE FM, SANFORD KK AND AARONSON SA. (1985). Neoplastic transformation of human epidermal Keratinocytes by Ad12-SV40 and Kirsten sarcoma viruses. *Science*, **227**, 1250–1252.

RHIM JS, FUJITA J, ARNSTEIN P AND AARONSON SA. (1986). Neoplastic conversion of human keratinocytes by adenovirus 12-SV40 virus and chemical carcinogens. *Science*, **232**, 385–388.

SCHEGEL R, PHELPS WC, ZHANG YL AND BARBOSA M. (1988). Quantitative keratinocyte assay detects two biological activities of human papilloma virus DNA and identifies viral types associated with cervical carcinoma. *EMBO J.*, **7**, 3181–3187.

SHIPLEY GD, CHILDS CB, VOLKENANT ME AND MOSES HL. (1984). Differential effects of epidermal growth factor, transforming growth factor and insulin on DNA and protein synthesis and morphology in serum free cultures on AKR-2B cells. *Cancer Res.*, **44**, 710–716.

SHIPLEY GD, PITTELKOW MR, WILLE JJ, SCOTT RE AND MOSES HL. (1986). Type beta transforming growth factor/growth inhibitor induces the reversible inhibition of normal human prokeratinocyte proliferation in serum free medium. *Cancer Res.*, **46**, 2068–2071.

STEINBERG ML AND DEFENDI V. (1979). Altered pattern of growth and differentiation in human keratinocytes infected by simian virus 40. *Proc. Natl Acad. Sci. USA*, **76**, 801–805.

STONER GD, KAIGHN E, REDDEL RR, RESAU JH, BOWMAN D, NAITO Z, MATSUKURA N, YOU, M, GALATI AJ AND HARRIS C. (1991). Establishment and characterization of SV40 T-antigen immortalized human esophageal epithelial cells. *Cancer Res.*, **51**, 365–371.

TSUDA T, TAHARA E, KAJIYAMA G, SAKAMOTO H, TERADA M AND SUGIMURA T. (1991). High incidence of coamplification of int-2 and hst-1 genes in human esophageal carcinomas. *Cancer Res.*, **49**, 5505–5508.

VAN DOREN K AND GLUZMAN Y. (1984). Efficient transformation of human fibroblasts by adenovirus-simian virus 40 recombinants. *Mol. Cell Biol.*, **4**, 1653–1656.

VAN DOREN K, HANAHAN D AND GLUZMAN Y. (1984). Infection of eukaryotic cells by helper independent recombinant adenovirus: early region is not obligatory for integration of viral DNA. *J. Virol.*, **50**, 606–614.

WANG DD, HONG JY, OUI SL, GAO H AND YANG CS. (1993). Accumulation of p53 protein in human esophageal precancerous lesions: a possible early biomarker for carcinogenesis. *Cancer Res.*, **53**, 1783–1787.

WHITE JA, CARTER SG, OZER HL AND BOYD AL. (1992). Cooperativity of SV40 T antigen and ras in progressive stages of transformation of human fibroblasts. *Exp. Cell Res.*, **203**, 157–163.

YUSPA S AND MORGAN D. (1981). Mouse skin cells resistant to terminal differentiation associated with initiation of carcinogenesis. *Nature*, **293**, 72–74.


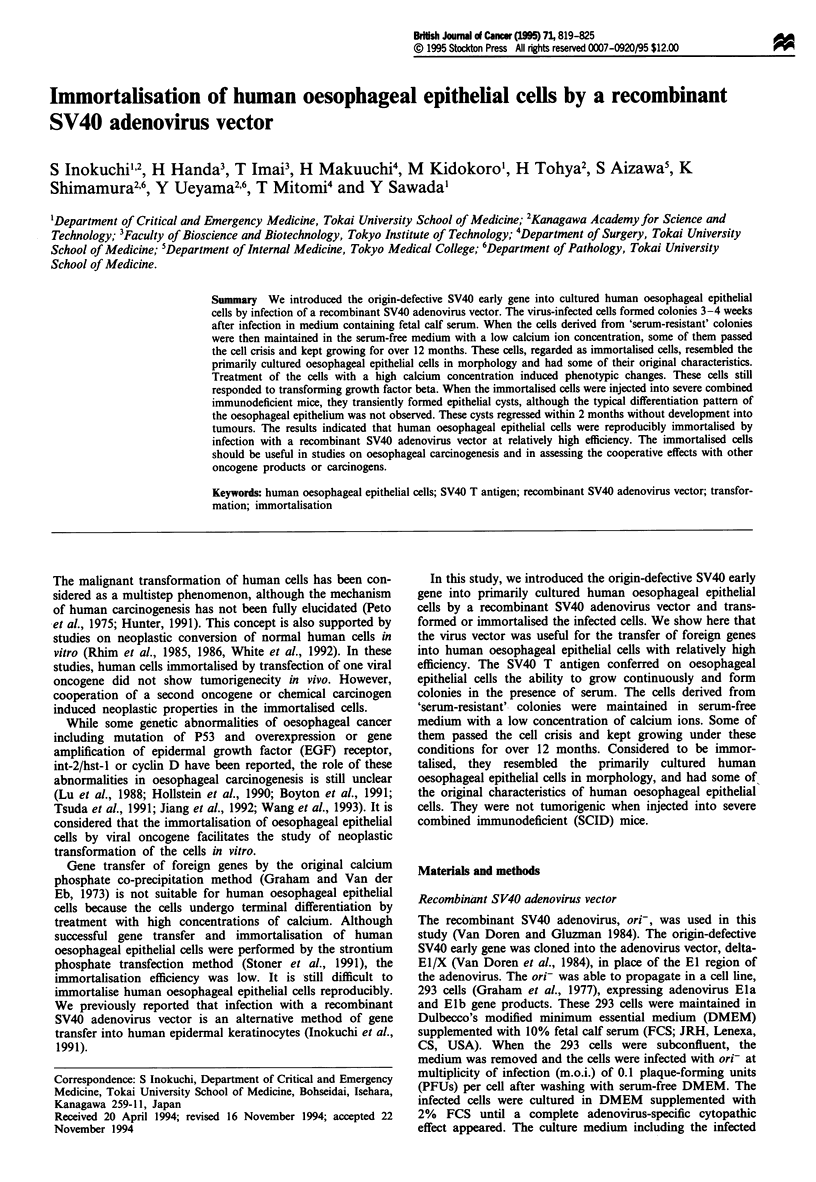

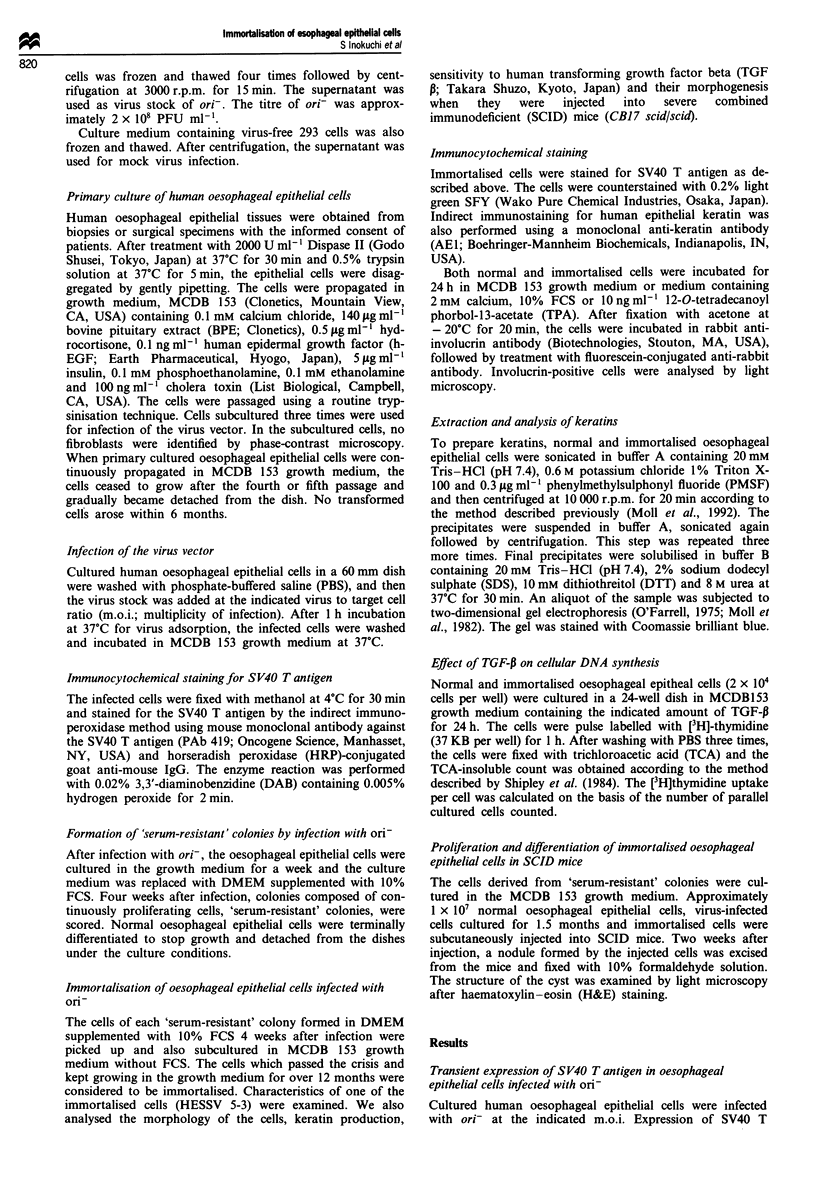

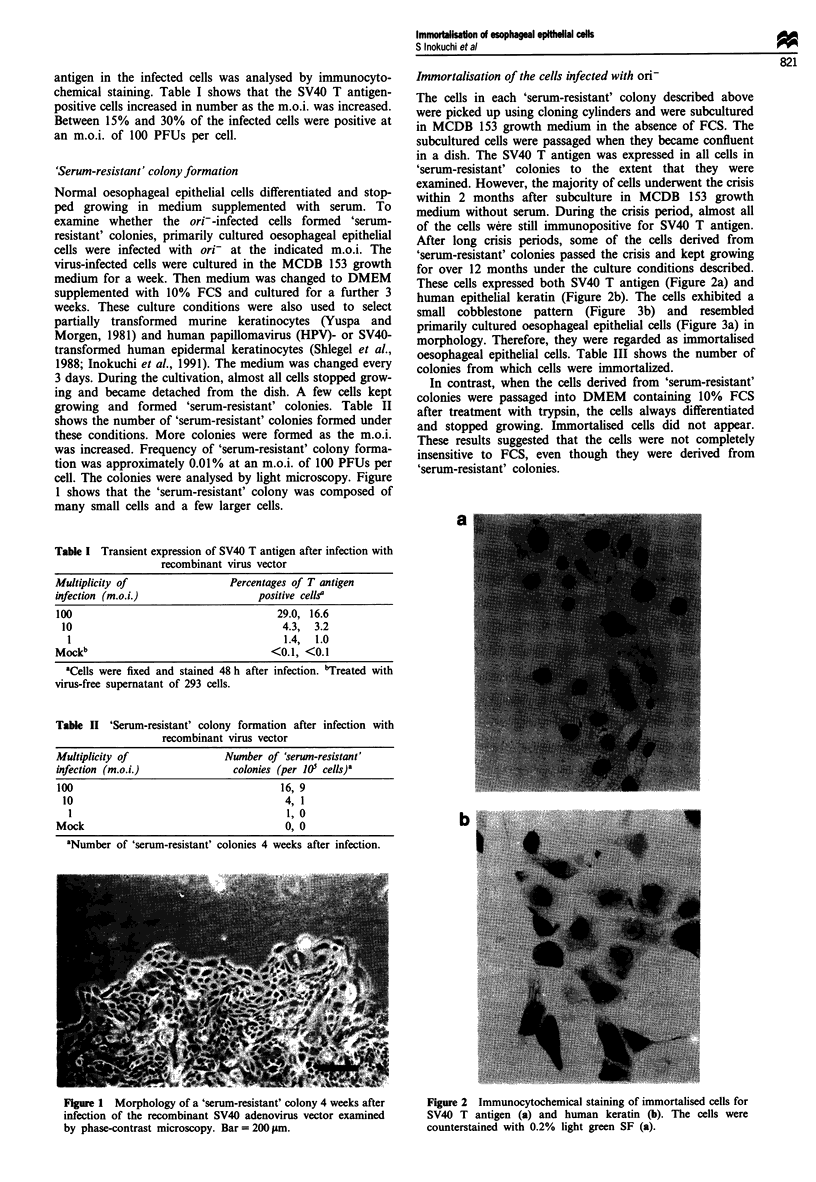

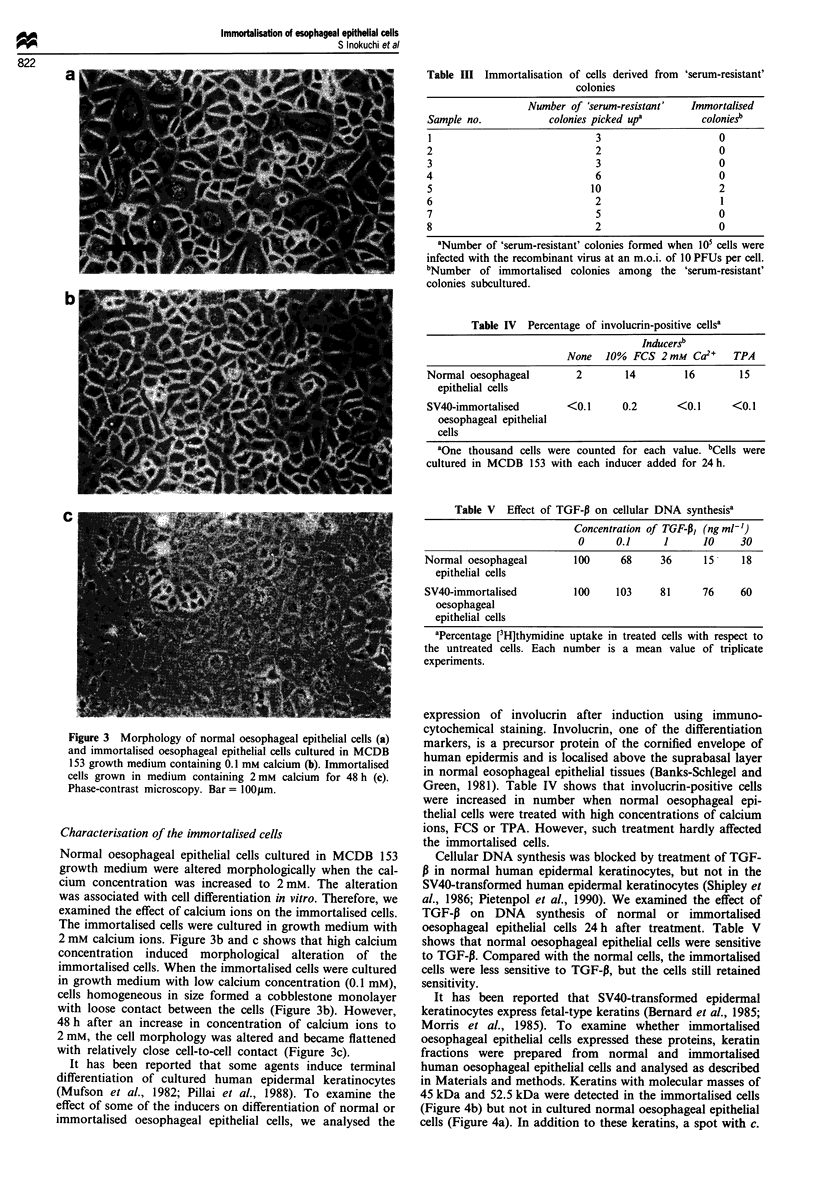

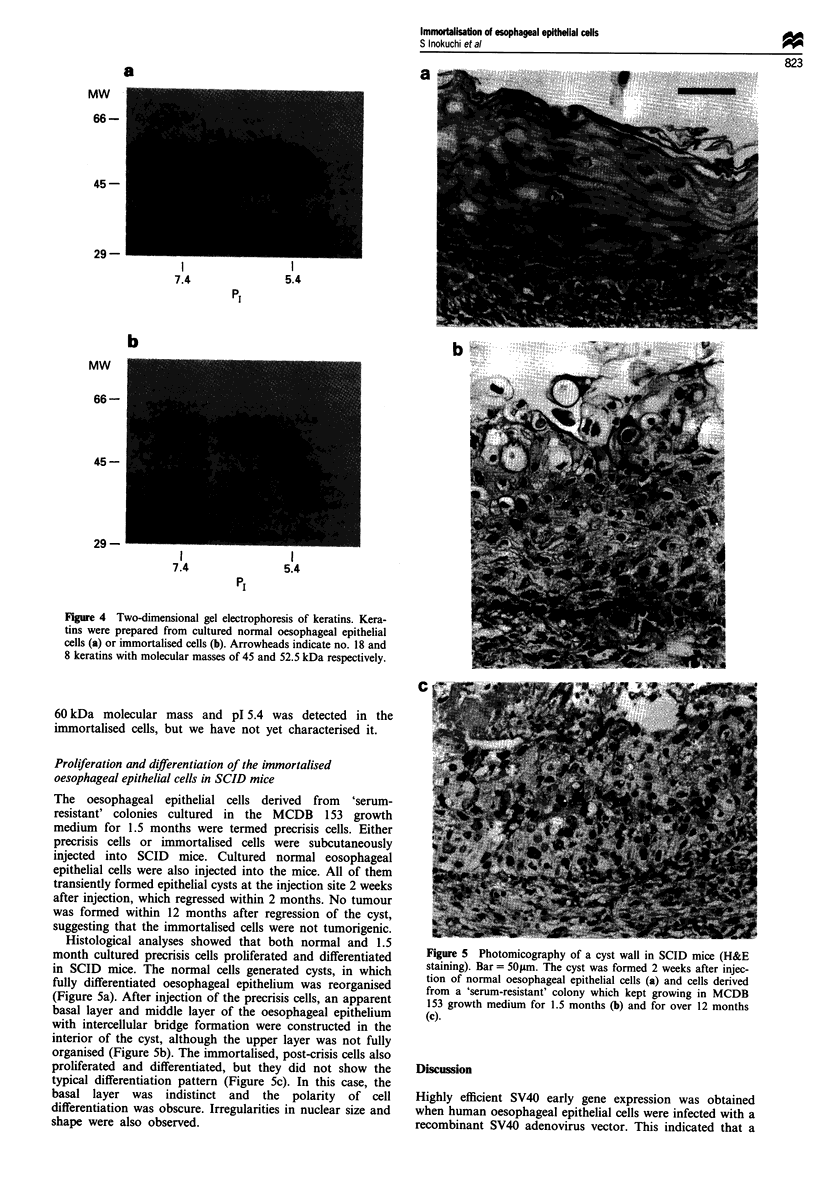

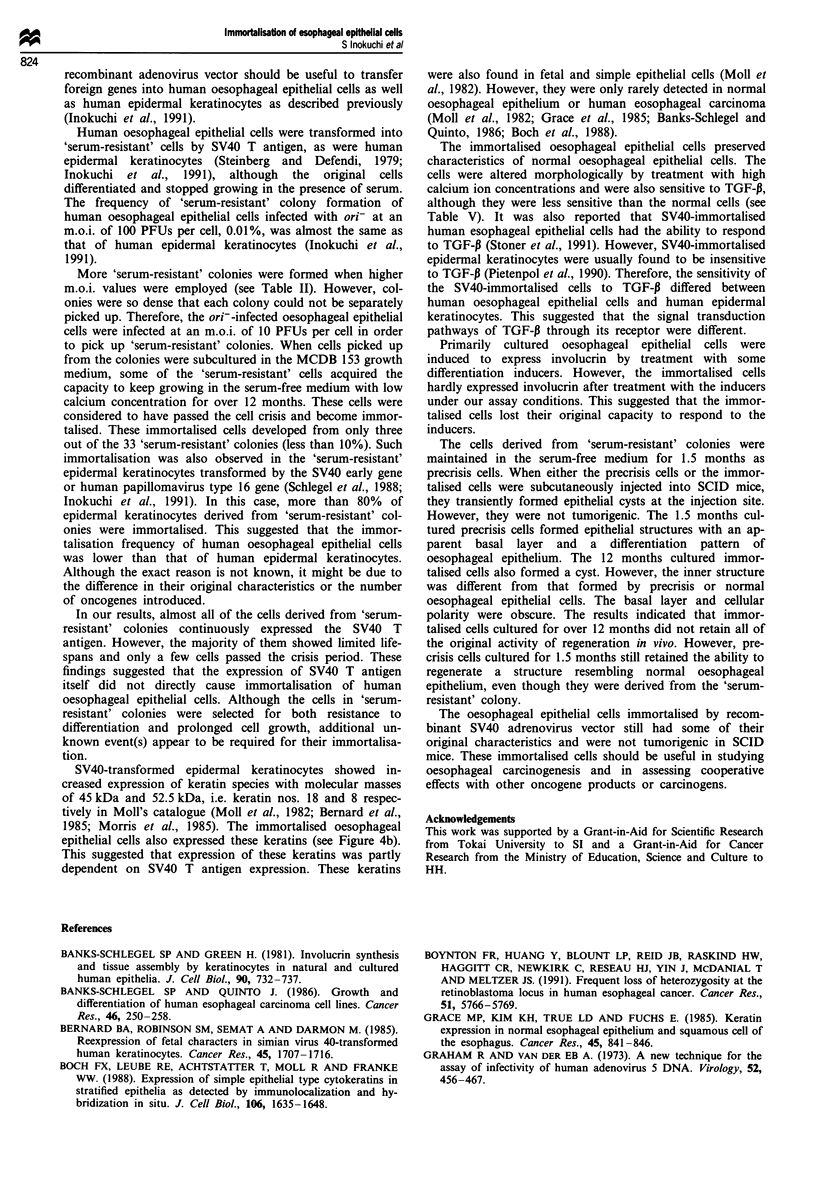

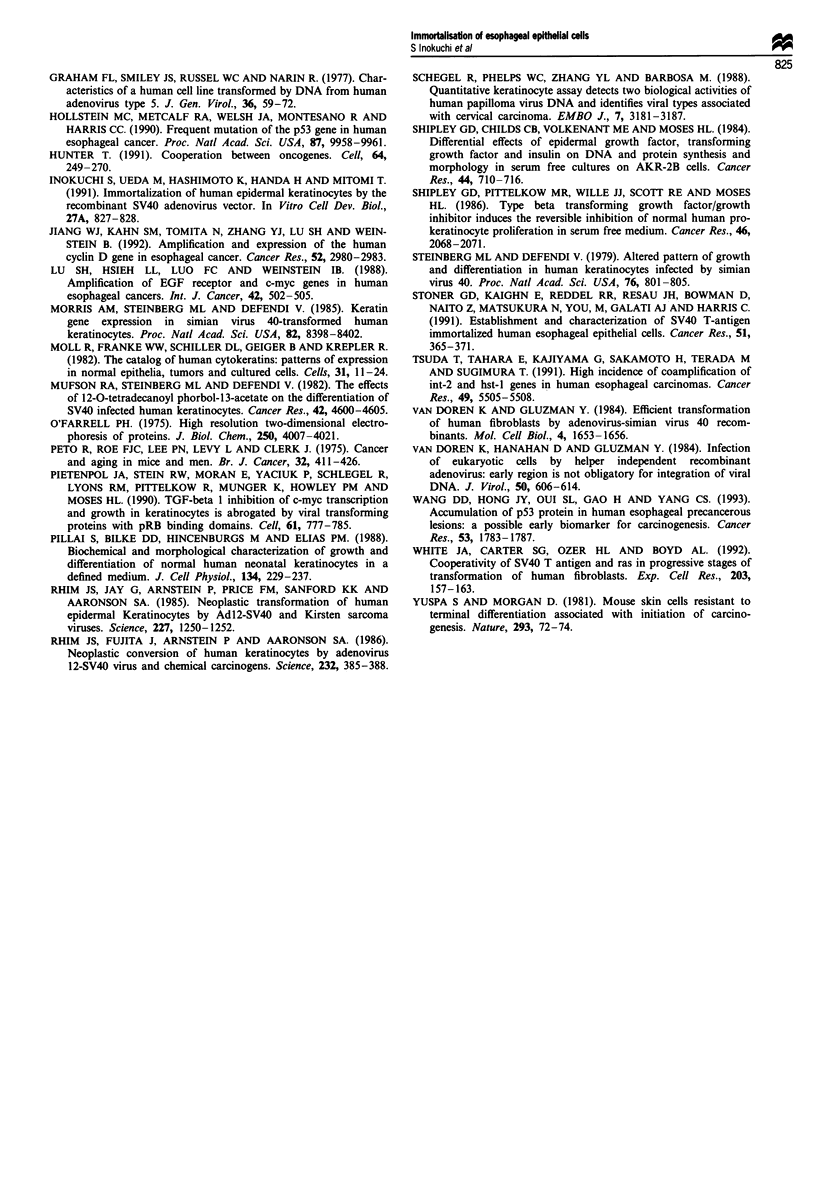


## References

[OCR_00747] Banks-Schlegel S. P., Quintero J. (1986). Growth and differentiation of human esophageal carcinoma cell lines.. Cancer Res.

[OCR_00744] Banks-Schlegel S., Green H. (1981). Involucrin synthesis and tissue assembly by keratinocytes in natural and cultured human epithelia.. J Cell Biol.

[OCR_00754] Bernard B. A., Robinson S. M., Semat A., Darmon M. (1985). Reexpression of fetal characters in simian virus 40-transformed human keratinocytes.. Cancer Res.

[OCR_00759] Bosch F. X., Leube R. E., Achtstätter T., Moll R., Franke W. W. (1988). Expression of simple epithelial type cytokeratins in stratified epithelia as detected by immunolocalization and hybridization in situ.. J Cell Biol.

[OCR_00766] Boynton R. F., Huang Y., Blount P. L., Reid B. J., Raskind W. H., Haggitt R. C., Newkirk C., Resau J. H., Yin J., McDaniel T. (1991). Frequent loss of heterozygosity at the retinoblastoma locus in human esophageal cancers.. Cancer Res.

[OCR_00770] Grace M. P., Kim K. H., True L. D., Fuchs E. (1985). Keratin expression in normal esophageal epithelium and squamous cell carcinoma of the esophagus.. Cancer Res.

[OCR_00775] Graham F. L., van der Eb A. J. (1973). A new technique for the assay of infectivity of human adenovirus 5 DNA.. Virology.

[OCR_00791] Hollstein M. C., Metcalf R. A., Welsh J. A., Montesano R., Harris C. C. (1990). Frequent mutation of the p53 gene in human esophageal cancer.. Proc Natl Acad Sci U S A.

[OCR_00795] Hunter T. (1991). Cooperation between oncogenes.. Cell.

[OCR_00801] Inokuchi S., Hashimoto K., Mitomi T., Ueda M., Handa H. (1991). Immortalization of human epidermal keratinocytes by the recombinant SV40 adenovirus vector.. In Vitro Cell Dev Biol.

[OCR_00805] Jiang W., Kahn S. M., Tomita N., Zhang Y. J., Lu S. H., Weinstein I. B. (1992). Amplification and expression of the human cyclin D gene in esophageal cancer.. Cancer Res.

[OCR_00809] Lu S. H., Hsieh L. L., Luo F. C., Weinstein I. B. (1988). Amplification of the EGF receptor and c-myc genes in human esophageal cancers.. Int J Cancer.

[OCR_00819] Moll R., Franke W. W., Schiller D. L., Geiger B., Krepler R. (1982). The catalog of human cytokeratins: patterns of expression in normal epithelia, tumors and cultured cells.. Cell.

[OCR_00825] Mufson R. A., Steinberg M. L., Defendi V. (1982). Effects of 12-O-tetradecanoylphorbol-13-acetate on the differentiation of simian virus 40-infected human keratinocytes.. Cancer Res.

[OCR_00829] O'Farrell P. H. (1975). High resolution two-dimensional electrophoresis of proteins.. J Biol Chem.

[OCR_00833] Peto R., Roe F. J., Lee P. N., Levy L., Clack J. (1975). Cancer and ageing in mice and men.. Br J Cancer.

[OCR_00838] Pietenpol J. A., Stein R. W., Moran E., Yaciuk P., Schlegel R., Lyons R. M., Pittelkow M. R., Münger K., Howley P. M., Moses H. L. (1990). TGF-beta 1 inhibition of c-myc transcription and growth in keratinocytes is abrogated by viral transforming proteins with pRB binding domains.. Cell.

[OCR_00844] Pillai S., Bikle D. D., Hincenbergs M., Elias P. M. (1988). Biochemical and morphological characterization of growth and differentiation of normal human neonatal keratinocytes in a serum-free medium.. J Cell Physiol.

[OCR_00856] Rhim J. S., Fujita J., Arnstein P., Aaronson S. A. (1986). Neoplastic conversion of human keratinocytes by adenovirus 12-SV40 virus and chemical carcinogens.. Science.

[OCR_00851] Rhim J. S., Jay G., Arnstein P., Price F. M., Sanford K. K., Aaronson S. A. (1985). Neoplastic transformation of human epidermal keratinocytes by AD12-SV40 and Kirsten sarcoma viruses.. Science.

[OCR_00861] Schlegel R., Phelps W. C., Zhang Y. L., Barbosa M. (1988). Quantitative keratinocyte assay detects two biological activities of human papillomavirus DNA and identifies viral types associated with cervical carcinoma.. EMBO J.

[OCR_00865] Shipley G. D., Childs C. B., Volkenant M. E., Moses H. L. (1984). Differential effects of epidermal growth factor, transforming growth factor, and insulin on DNA and protein synthesis and morphology in serum-free cultures of AKR-2B cells.. Cancer Res.

[OCR_00875] Shipley G. D., Pittelkow M. R., Wille J. J., Scott R. E., Moses H. L. (1986). Reversible inhibition of normal human prokeratinocyte proliferation by type beta transforming growth factor-growth inhibitor in serum-free medium.. Cancer Res.

[OCR_00881] Steinberg M. L., Defendi V. (1979). Altered pattern of growth and differentiation in human keratinocytes infected by simian virus 40.. Proc Natl Acad Sci U S A.

[OCR_00886] Stoner G. D., Kaighn M. E., Reddel R. R., Resau J. H., Bowman D., Naito Z., Matsukura N., You M., Galati A. J., Harris C. C. (1991). Establishment and characterization of SV40 T-antigen immortalized human esophageal epithelial cells.. Cancer Res.

[OCR_00894] Tsuda T., Tahara E., Kajiyama G., Sakamoto H., Terada M., Sugimura T. (1989). High incidence of coamplification of hst-1 and int-2 genes in human esophageal carcinomas.. Cancer Res.

[OCR_00899] Van Doren K., Gluzman Y. (1984). Efficient transformation of human fibroblasts by adenovirus-simian virus 40 recombinants.. Mol Cell Biol.

[OCR_00904] Van Doren K., Hanahan D., Gluzman Y. (1984). Infection of eucaryotic cells by helper-independent recombinant adenoviruses: early region 1 is not obligatory for integration of viral DNA.. J Virol.

[OCR_00908] Wang L. D., Hong J. Y., Qiu S. L., Gao H., Yang C. S. (1993). Accumulation of p53 protein in human esophageal precancerous lesions: a possible early biomarker for carcinogenesis.. Cancer Res.

[OCR_00914] White J. A., Carter S. G., Ozer H. L., Boyd A. L. (1992). Cooperativity of SV40 T antigen and ras in progressive stages of transformation of human fibroblasts.. Exp Cell Res.

[OCR_00920] Yuspa S. H., Morgan D. L. (1981). Mouse skin cells resistant to terminal differentiation associated with initiation of carcinogenesis.. Nature.

